# PHLDA2 is critical for p53-mediated ferroptosis and tumor suppression

**DOI:** 10.1093/jmcb/mjae033

**Published:** 2024-08-29

**Authors:** Xin Yang, Wei Gu

**Affiliations:** Institute for Cancer Genetics, and Herbert Irving Comprehensive Cancer Center, Vagelos College of Physicians & Surgeons, Columbia University, New York, NY 10032, USA; Jiangsu Key Laboratory of Infection and Immunity, The Institutes of Biology and Medical Sciences, Suzhou Medical College, Soochow University, Suzhou 215123, China; Institute for Cancer Genetics, and Herbert Irving Comprehensive Cancer Center, Vagelos College of Physicians & Surgeons, Columbia University, New York, NY 10032, USA; Department of Pathology and Cell Biology, Vagelos College of Physicians & Surgeons, Columbia University, New York, NY 10032, USA

**Keywords:** PHLDA2, ROS, ferroptosis, cancer, tumor suppression, p53

**Dear Editor**,

Inactivation of the tumor suppressor p53 is a pivotal event in the formation of most human cancers. Dissecting the precise mechanism of p53 in tumor suppression contributes to the development of a better strategy for cancer therapy. The canonical activities of p53, such as cell cycle arrest, senescence, and apoptosis, are well accepted as the major checkpoints in stress responses and tumor suppression. However, accumulating evidence suggests that p53 still exerts tumor-suppressive effects even without these canonical abilities (Brady et al., 2011; Li et al., 2012). We and others recently found that p53 plays an important role in promoting ferroptotic responses through transcriptional regulation (Liu and Gu, 2022). For example, p53 transcriptionally represses SLC7A11, the cystine–glutamate antiporter, to sensitize tumor cells to ferroptosis in a glutathione-dependent manner (Jiang et al., 2015; Leu et al., 2019). On the other hand, p53 inhibits VKORC1L1 to restrain the content of reduced vitamin K, a newly identified type of radical-trapping antioxidant, to promote ferroptosis (Yang et al., 2023).

Interestingly, although p53 activation is able to modulate the ferroptotic response induced by GPX4 inhibitors, p53-mediated tumor suppression is more closely linked with a distinct ferroptosis pathway upon high levels of reactive oxygen species (ROS) in the absence of common ferroptosis inducers (Jiang et al., 2015; Liu and Gu, 2022). Indeed, the lipoxygenase ALOX12 was identified as a critical factor in ROS-induced ferroptosis during p53-mediated tumor suppression (Chu et al., 2019), and our recent study further showed that PHLDA2 is able to recognize phosphatidic acid and induce its peroxidation by recruiting ALOX12 for tumor suppression (Yang et al., 2024). By using both immunodeficient and immunocompetent mouse tumor models, we found that PHLDA2 is crucial for tumor suppression by inducing ferroptosis naturally *in vivo* without any drug treatment from common ferroptosis inducers.

Consequently, it is interesting to examine whether PHLDA2 is directly involved in p53-mediated tumor suppression by inducing ferroptosis. To this end, we first examined whether PHLDA2 is directly involved in modulating p53-dependent ferroptotic response upon high levels of ROS. As shown in Figure 1A, CRISPR–Cas9-mediated knockout of PHLDA2 in human osteosarcoma U2OS cells had no obvious effect on p53 levels or p53 transcriptional ability, suggesting that PHLDA2 is not directly involved in modulating p53-dependent transcriptional activity. Moreover, activation of p53 upon Nutlin-3 treatment sensitized U2OS cells to ferroptosis in the presence of the ROS-generating reagent tert-butyl hydroperoxide (TBH, 100 µM) (Figure 1B and C, *P* = 0.003), which was largely abrogated in isogeneic PHLDA2^–/–^ U2OS cells (Figure 1B and C, *P* = 0.76), suggesting that PHLDA2 is crucial for p53-mediated ferroptosis. Furthermore, PHLDA2 was found to be critical for p53-mediated ferroptotic responses upon the treatment of TBH or another ROS-generating reagent cumene hydroperoxide (CMH) at various concentrations (Supplementary Figure S1A and B, *P* < 0.05). To corroborate these findings, we knocked out PHLDA2 in human breast cancer MCF7 cells (Figure 1D). Again, loss of PHLDA2 did not affect p53 levels or p53 transcription ability but abrogated p53-mediated ferroptotic cell death upon TBH or CMH treatment (Figure 1D–F; Supplementary Figure S1C and D, *P *<* *0.05). Taken together, our results demonstrate that PHLDA2 is required for p53-mediated ferroptosis in response to ROS-induced stress.

**Figure 1:**
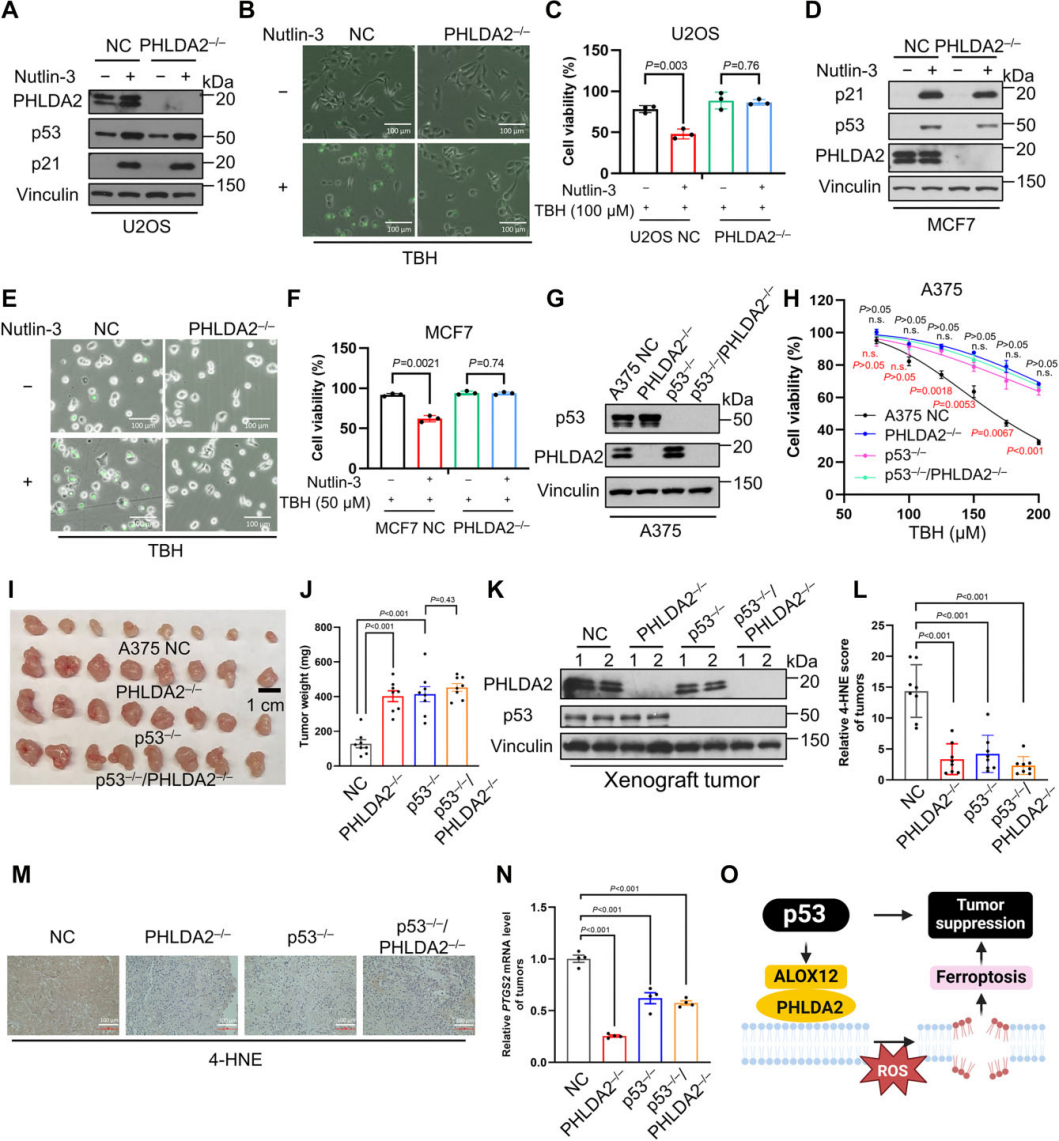
PHLDA2 is critical for p53-mediated ferroptosis. (**A**–**C**) Negative control (NC) or PHLDA2^–/–^ U2OS cells were treated with or without Nutlin-3 (5 µM) for 24 h. (**A**) Western blot analysis for the indicated proteins. (**B**) Representative phase-contrast SYTOX staining images after TBH treatment for 8 h. Scale bar, 100 µm. (**C**) Cell viability after 100 µM TBH treatment for 8 h. (**D**–**F**) NC or PHLDA2^–/–^ MCF7 cells were treated with or without Nutlin-3 (5 µM) for 24 h. (**D**) Western blot analysis for the indicated proteins. (**E**) Representative phase-contrast SYTOX staining images after TBH treatment for 8 h. Scale bar, 100 µm. (**F**) Cell viability after 50 µM TBH treatment for 8 h. (**G** and **H**) Western blot analysis of NC, PHLDA2^–/–^, p53^–/–^, or p53^–/–^/PHLDA2^–/–^ A375 cells. (**G**) Western blot analysis for the indicated proteins. (**H**) Cell viability after TBH treatment for 8 h. *P*-values shown in black indicate the comparison between PHLDA2^–/–^ cells with and without Nutlin-3 treatment; *P*-values shown in red indicate the comparison between NC with and without Nutlin-3 treatment. n.s. represents not significant. (**I**–**N**) NC, PHLDA2^–/–^, p53^–/–^, or p53^–/–^/PHLDA2^–/–^ A375 cells were subcutaneously injected into BALB/c nude mice. (**I**) Harvested xenograft tumors. Scale bar, 1 cm. (**J**) Tumor weights. (**K**) Western blot analysis for the indicated proteins. (**L**) Relative 4-HNE score of tumors determined by immunochemistry. (**M**) Representative immunochemistry images of 4-HNE staining. Magnification, 20×; scale bar, 100 µm. (**N**) Relative *PTGS2* mRNA level of tumors determined by quantitative PCR. (**O**) Work model demonstrates the involvement of PHLDA2 in p53-mediated tumor suppression through modulating ferroptosis. Data are mean ± SEM of *n* = 8 independent tumors per group (**J**) or mean ± SD of *n* = 3 (**C**, **F**, and **H**), *n* = 8 (**L**), or *n* = 4 (**N**) independent repeats. All cell viability assays were performed by using at least three biological replications. *P*-values were calculated using unpaired, two-tailed Student's *t*-test.

Next, we generated isogenic A375-derived PHLDA2^–/–^, p53^–/–^, and p53^–/–^/PHLDA2^–/–^ cell lines (Figure 1G). As expected, p53-mediated ferroptosis was readily induced in control A375 cells upon high levels of ROS (Figure 1H, *P*-values in red) but largely abolished in PHLDA2^–/–^ cells (Figure 1H, *P*-values in black). Conversely, in isogenic p53-null A375 cells, loss of PHLDA2 did not affect cell viability upon high levels of ROS (Figure 1H), suggesting that PHLDA2 is specifically required for p53-dependent ferroptotic response even without Nutlin-3 treatment. Then, we injected these isogenic cell lines subcutaneously into BALB/c nude mice for tumor growth assays. Likewise, either loss of PHLDA2 (control A375 vs. PHLDA2^–/–^) or p53 inactivation (control A375 vs. p53^–/–^) significantly promoted A375 xenograft tumor growth, while loss of PHLDA2 in p53-null A375 cells failed to further promote tumor growth (p53^–/–^ vs. p53^–/–^/PHLDA2^–/–^) (Figure 1I–K). Moreover, the levels of 4-HNE and *PTGS2,* two well-known ferroptosis markers, both significantly decreased upon loss of PHLDA2 or p53 (Figure 1L–N). These data indicate that PHLDA2 is critical for p53-mediated tumor suppression through ferroptosis.

Our recent study detected *in vivo* PHLDA2-mediated ferroptosis in an ACSL4-independent manner in the absence of any common ferroptosis inducers, suggesting the occurrence of ferroptosis under normal physiological settings without any treatment (Yang et al., 2024). Moreover, the tumor-suppressive role of PHLDA2 has been implicated in different mouse tumor models, including the genetically well-defined lymphoma model and the pharmacologically induced liver cancer model (Yang et al., 2024). Taken together, these data demonstrate that PHLDA2-dependent ferroptosis, but not ACSL4-dependent ferroptosis, occurring naturally *in vivo* upon ROS-induced stress, plays a critical role in p53-mediated tumor suppression (Figure 1O). Future investigation is required to further dissect the regulation of the p53–PHLDA2 axis to activate ferroptosis-mediated tumor suppression for a potential strategy in cancer therapy.

*[Supplementary material is available at *Journal of Molecular Cell Biology* online. The content is solely the responsibility of the authors and does not necessarily represent the official views of the National Institutes of Health. The work from W.G.’s lab was supported by the National Cancer Institute of the National Institutes of Health under Award R35CA253059, RO1CA258390, and R01CA254970. X.Y. and W.G. conceived and designed the experiments. X.Y. and W.G. acquired methodology and data. X.Y. conducted analysis and interpretation of data. X.Y. and W.G wrote the manuscript*.]

## Supplementary Material

mjae033_Supplemental_File
