# Supplementary material for: PHLDA2 is critical for p53-mediated ferroptosis and tumor suppression
Source: J Mol Cell Biol. 2024 Aug 29;16(7):mjae033. doi: 10.1093/jmcb/mjae033 (PMC11713609; doi:10.1093/jmcb/mjae033)
Supplement: mjae033_Supplemental_File [file mjae033_supplemental_file.pdf]

Supplementary material

Supplementary Materials and methods

Cell culture and reagents

Human breast cancer MCF-7 (p53 wildtype), human bone osteosarcoma U2OS (p53 wildtype), and human melanoma A375 (p53 wildtype) were previously obtained from ATCC. Cells were cultured with DMEM supplemented with 10% (*v*/*v*) FBS (Gibco), 100 units/ml penicillin, and 100 μg/ml streptomycin (Gibco) in a humidified incubator at 37°C and 5% CO_2. Cells were treated with tert-Butyl hydroperoxide (TBH; Sigma-Aldrich, 458139), Cumene hydroperoxide (CMH; Sigma-Aldrich, 247502), and Nutlin-3 (Sigma-Aldrich, N6287) as indicated concentration for indicated time.

All cell lines have been regularly tested to be negative for mycoplasma contamination every month. No cell lines used in this work were listed in the ICLAC database. The cell lines were freshly thawed from the purchased seed cells and were cultured for no more than one month. The morphology of cell lines was checked before the experiments and compared with the ATCC cell line image to avoid cross-contamination or misuse of cell lines.

Cell viability assay

Unless otherwise specified, cells were seeded in white, sterile, and tissue culture-treated opaque 96-well microplates (PerkinElmer) at 5 × 10^3 cells per well. After ~18 h (overnight), the cells were pre-treated with indicated compounds for the indicated time before further treatment or directly treated with indicated compounds at indicated concentrations for the indicated time. There were three biological replicates per condition. Cellular ATP levels were quantified using CellTiter-Glo 2.0 reagent (Promega) following the manufacturer's instructions by GloMax Discover Microplate Reader (Promega). Relative cell viability was measured in comparison to the relative untreated condition. Nonlinear regression analysis of the mean ± SD; $n = 3$ biological replicates of each data point was used to measure the fit curves of cell viability by GraphPad Prism 8.0.

Cell death imaging

Cells were seeded in a 12-well plate (Corning) at 4 × 10^4 cells per well. After ~18 h (overnight), the cells were pre-treated with indicated compounds for the indicated time before further treatment or directly treated with indicated compounds at indicated concentrations for the indicated time. Then, 30 nM SYTOX green dead cell stain (Invitrogen, S34860) was added into plates and incubated for 1 h at 37°C. At least three randomly

chosen bright-fields and FITC fluorescence-fields were captured by microscopy (Olympus, IX51). Living cells were free of green staining and dead cells were green-stained.

Western blot and antibodies

Cells were lysed with Flag lysis buffer [50 mM Tris–HCl (pH 7.9), 137 mM NaCl, 1% Triton X-100, 0.2% Sarkosyl, 1 mM NaF, 1 mM Na_3VO_4, and 10% glycerol] containing protease inhibitor cocktail, 1 mM DTT, and 1 mM PMSF. The same amount of protein from different experiment groups was quantified and detected by western blot analysis. Western blotting was conducted for protein analysis according to standard methods with 4%–20% pre-cast SDS–PAGE gel (Invitrogen, XP0420). Commercial antibodies were used: PHLDA2 (ThermoFisher Scientific, PA5-92845, 1:1000 dilution), Vinculin (Sigma-Aldrich, V9131, 1:10000 dilution), p53 (DO-1) (Santa Cruz Biotechnology, sc-126, 1:5000 dilution), and p21 (Cell Signaling Technology, 2947, 1:1000 dilution). Horseradish peroxidase-conjugated goat anti-mouse and anti-rabbit secondary antibodies (Jackson ImmunoResearch) were used.

Reverse transcription–quantitative PCR (RT–qPCR)

Total RNA of tumor tissue was isolated using TRIzol reagent (Invitrogen) according to the manufacturer’s instructions. cDNA was reversed by SuperScript IV VILO Master Mix (Invitrogen). qPCR was then performed using SYBR Green Master Mix (Invitrogen) to detect mRNA expression levels of indicated genes. The expression levels of target genes were normalized by that of *GAPDH*. The primers for human *GAPDH* and *PTGS2* genes were: *PTGS2* forward 5’-CTTCACGCATCAGTTTTTCAAG-3’, *PTGS2* reverse 5’-TCACCGTAAATATGATTTAAGTCCAC-3’; *GAPDH* forward 5’-GAAGGTGAAGGTCGGAGTC-3’, *GAPDH* reverse 5’-GAAGATGGTGATGGGATTTC-3’.

CRISPR–Cas9 system-mediated gene ablation

p53 knockout cells were previously described (Chen et al., 2021). To generate A375 $p53^{-/-}$/$PHLDA2^{-/-}$ double-null cells, A375 $p53^{-/-}$ cells were co-transfected with PHLDA2 gene TrueGuide Synthetic guide RNA (Invitrogen, A35533) or Negative Control non-targeting RNA (Invitrogen, A35526) with TrueCut Cas9 protein v2 (Invitrogen, A36498) by Lipofectamine CRISPRMAX Transfection Reagent (Invitrogen, CMAX00015). After 48 h, pooled cells were seeded for cell viability or western blot validation, and the rest of the cells were split into single cells for colony culture and further analysis. The catalog numbers of PHLDA2 sgRNA purchased from Invitrogen were CRISPR676994_SGM and CRISPR676991_SGM.

Immunohistochemical (IHC) staining

Tumor samples were fixed in 10% formalin for 24 h and 70% ethanol before the standard dehydration processing for preparing the paraffin blocks. Paraffin blocks were sectioned at 4 μM thickness for IHC staining. Tissue sections were deparaffinized with xylene and followed with gradient ethanol (100%, 95%, 90%, 80%, and 70%). After rinsing with deionized water for 5 min, the sections were incubated in 3% hydrogen peroxide for 20 min to eliminate endogenous peroxidase. To retrieve antigen, the sections were incubated in 10 mM sodium citrate buffer (pH 6.0) at 100°C for 20 min. Then, the specimens were washed with PBS three times and incubated with 4-HNE antibody (Abcam, ab46545, 1:200) at 4°C overnight. The next day, the specimens were rinsed with PBS three times, followed by staining with the ImmPRESS HRP Horse Anti-Rabbit IgG Polymer Detection Kit (MP-7401, Vector laboratory). Finally, ImmPACT DAB Substrate Kit (SK-4105, Vector laboratory) was used to detect the signal. The IHC images were photographed in microscopy (Eclipse Ni-U, Nikon), and staining scores for each specimen were determined by calculating the relative IOD/Area ratio using Image-pro plus software.

Xenografts experiments

Six-week-old female nude mice (Nu/Nu, Charles River) were used for xenograft experiments. The mice were housed in a temperature-controlled room (65°F–75°F) with 40%–60% humidity, with a light/dark cycle of 12 h/12 h. Animal experiments were approved by the Institutional Animal Care and Use Committee (IACUC) at Columbia University under the supervision of the Institute of Comparative Medicine (ICM). Randomization of animals was performed before the xenograft experiments. The tumors in the xenograft experiments did not exceed the limit for tumor burden (10% of total body weight or 2 cm in diameter).

A total of 3.0×10^6 A375 NC, PHLDA2$^{-/-}$, p53$^{-/-}$, or p53$^{-/-}$/PHLDA2$^{-/-}$ cells resuspended in sterile PBS were mixed with Matrigel (Corning) at a 1:1 (*v*/*v*) ratio and injected subcutaneously into nude mice. Mice were euthanized 3 weeks after injection, and tumors were dissected and weighed.

Quantification, statistics, and reproducibility

Experiments independently repeated at least three times were stated in figure legends. A two-tailed unpaired Student’s *t*-test by GraphPad Prism 8.0 was done for the statistical analyses without specific statements. Data presented in the figures were shown with an error of the mean (mean ± SD) without specific statements. $P < 0.05$ was considered statistically significant between groups. Data were graphed using GraphPad Prism 8.0.

Supplementary Reference

Chen, D., Chu, B., Yang, X., et al. (2021). iPLA2β-mediated lipid detoxification controls p53-driven ferroptosis independent of GPX4. Nat. Commun. *12*, 1-15.

Supplementary Figure

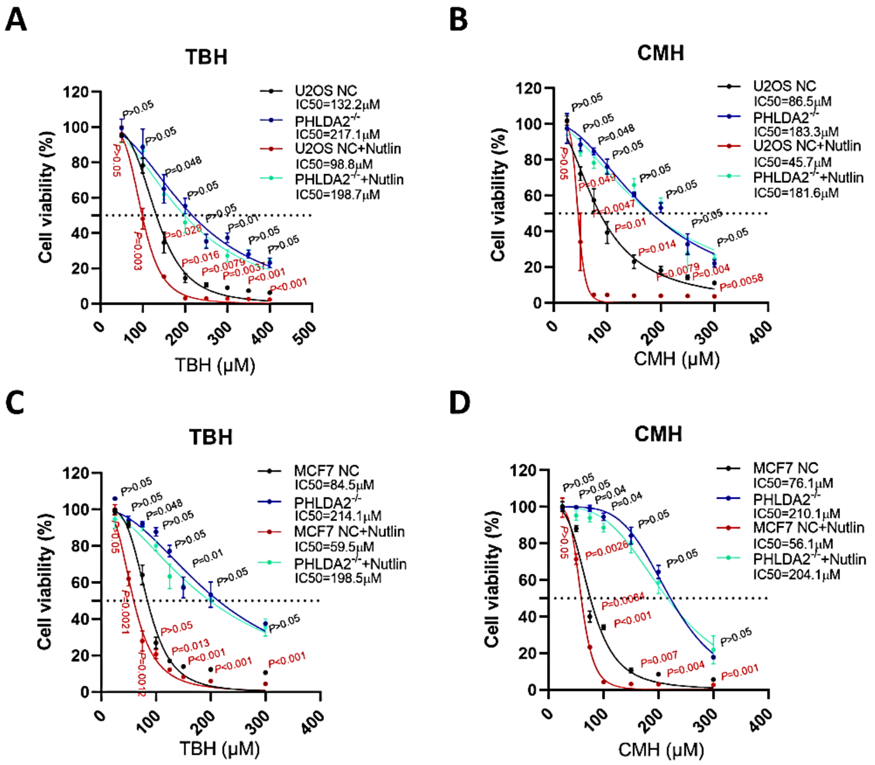

Supplementary Figure S1 PHLDA2 is critical for p53-mediated ferroptosis.

(**A**) Cell viability of NC or PHLDA2$^{-/-}$ U2OS cells without or with 24 h Nutlin-3 (5 µM) pre-treatment and then under TBH treatment for 8 h.

(**B**) Cell viability of NC or PHLDA2$^{-/-}$ U2OS cells without or with 24 h N utlin-3 (5 µM) pre-treatment and then under CMH treatment for 6 h.

(**C**) Cell viability of NC or PHLDA2$^{-/-}$ MCF7 cells without or with 24 h Nutlin-3 (5 µM) pre-treatment and then under TBH treatment for 8 h.

(**D**) Cell viability of NC or PHLDA2$^{-/-}$ MCF7 cells without or with 24 h Nutlin-3 (5 µM) pre-treatment and then under CMH treatment for 6 h.

Data are presented as mean ± SD of *n* = 3 independent repeats. *P*-values were calculated using unpaired, two-tailed Student’s *t*-test. *P*-values shown in black color indicate the comparison between PHLDA2$^{-/-}$ cells with and without Nutlin-3 treatment; *P*-values shown in red color indicate the comparison between NC with and without Nutlin-3 treatment. All cell viability assays were performed using at least three biological replications.
